# Large-Scale Networks for Auditory Sensory Gating in the Awake Mouse

**DOI:** 10.1523/ENEURO.0207-19.2019

**Published:** 2019-09-09

**Authors:** Abbas Khani, Florian Lanz, Gerard Loquet, Karl Schaller, Christoph Michel, Charles Quairiaux

**Affiliations:** 1 Functional Brain Mapping Lab., Department of Basic Neurosciences, University of Geneva, CH-1211 Geneva, Switzerland; 2 Department of Clinical Medicine, Aalborg University, 9000 Aalborg, Denmark; 3 Division of Neurosurgery, University Hospitals of Geneva, University of Geneva, CH-1211 Geneva, Switzerland; 4 Centre for Biomedical Imaging (CIBM), CH-1015 Lausanne, Switzerland

**Keywords:** auditory cortex, brain state, cochlear nucleus, inferior colliculus, large-scale networks, sensory gating

## Abstract

The amplitude of the brain response to a repeated auditory stimulus is diminished as compared to the response to the first tone (T1) for interstimulus intervals (ISI) lasting up to hundreds of milliseconds. This adaptation process, called auditory sensory gating (ASG), is altered in various psychiatric diseases including schizophrenia and is classically studied by focusing on early evoked cortical responses to the second tone (T2) using 500-ms ISI. However, mechanisms underlying ASG are still not well-understood. We investigated ASG in awake mice from the brainstem to cortex at variable ISIs (125–2000 ms) using high-density EEG and intracerebral recordings. While ASG decreases at longer ISIs, it is still present at durations (500–2000 ms) far beyond the time during which brain responses to T1 could still be detected. T1 induces a sequence of specific stable scalp EEG topographies that correspond to the successive activation of distinct neural networks lasting about 350 ms. These brain states remain unaltered if T2 is presented during this period, although T2 is processed by the brain, suggesting that ongoing networks of brain activity are active for longer than early evoked-potentials and are not overwritten by an upcoming new stimulus. Intracerebral recordings demonstrate that ASG is already present at the level of ventral cochlear nucleus (vCN) and inferior colliculus and is amplified across the hierarchy in bottom-up direction. This study uncovers the extended stability of sensory-evoked brain states and long duration of ASG, and sheds light on generators of ASG and possible interactions between bottom-up and top-down mechanisms.

## Significance Statement

Stimulus-evoked responses of neurons fade rapidly and last usually up to around 100 ms in small brains such as the mice brain. The brain also attenuates the response to the second of a pair of stimuli (e.g., auditory), a phenomenon that is called sensory gating and is impoverished in schizophrenia patients. Here, first, we demonstrate that sensory gating is present in ventral cochlear nucleus (vCN), the first station of auditory pathway in the CNS, and is hierarchically organized such that sensory gating is amplified as the signal travels from early brain regions to higher order processing areas. Second, we show that brain networks are active for longer than early evoked-potentials highlighting the importance of often-neglected late components of brain response to sensory stimuli.

## Introduction

In the auditory system, a single repetition of a tone is enough for the brain to diminish the amplitude of the neural response to the second tone (T2). This adaptive property, called auditory sensory gating (ASG), has been detected in many brain regions in human and animals including auditory cortices (Grunwald et al., 2003; Wehr and Zador, 2005; Kurthen et al., 2007; Mayer et al., 2009), prefrontal areas (Alho et al., 1994; Mears et al., 2006; Williams et al., 2011), parietal areas (Grunwald et al., 2003; Knott et al., 2009), amygdala (Cromwell et al., 2005), and hippocampus (Grunwald et al., 2003; Witten et al., 2016). In previous works (Brosch and Schreiner, 1997; Wehr and Zador, 2005), forward masking, forward inhibition and two-tone interaction have almost interchangeably been used to describe sensory gating or very similar phenomena. Sensory gating is thought to be a fundamental mechanism that contributes to information processing by filtering extraneous sensory inputs (Qi et al., 2015), by minimizing surprise based on sensory inputs prediction (Rao and Ballard, 1999; Friston, 2018; Giraud and Arnal, 2018) or by serving as an energy saving system since the brain must adopt energy efficient mechanisms (Niven and Laughlin, 2008). Impairment in neuronal adaptation to successively presented auditory stimuli is manifest in schizophrenia patients and may represent a core physiologic dysfunction (Adler et al., 1982; Grunwald et al., 2003; Javitt and Sweet, 2015).

Several mechanisms may be responsible for ASG, from local short-term synaptic mechanisms in the auditory cortices (Wehr and Zador, 2005; Christianson et al., 2011) to larger-scale bottom-up or top-down modulations, and these mechanisms may act at different time-scales in the course of different durations of the interstimulus interval (ISI). Ascending projections from brainstem reticular neurons to the medial septal nucleus have been proposed as a bottom-up mechanism that would promote inhibition in the hippocampus, thalamus and neocortex (Javitt and Freedman, 2015). On the other hand, top-down mechanisms have been hypothesized in which ASG would be modulated by corticofugal projections to reticular thalamus inhibitory circuits (Yu et al., 2004; Duque et al., 2014) or by inhibitory modulations in auditory cortices projecting from the prefrontal cortex (Alho et al., 1994; Grunwald et al., 2003; Mears et al., 2006; Williams et al., 2011). Still, as simple the ASG phenomenon may seem, the neural correlates of ASG and especially its connections to higher order brain functions remain poorly understood.

In which brain region within the auditory network is ASG first detectable? How long after the first tone (T1) is the response to the T2 still gated? What are the differences between ASG following short and long ISIs in local brain areas and global brain states? As yet, we do not have clear responses to these questions. Indeed, most ASG studies used a single ISI of 500 ms and focused their analyses on specific surface EEG components or on early stimulus-evoked responses in local brain areas. For a better understanding of the mechanisms underpinning ASG, three points are important to be considered. First, we must study the dynamics of ASG in human and in awake animal models from short to long ISI as the adaptive mechanisms may be different for different ISI durations. Second, the effects of auditory stimuli and ASG should be examined beyond the initial evoked response, i.e., further during the ISI. Finally, ASG should be studied at large-scale level as auditory stimuli activate a large set of neural networks and we hypothesized that they potentially exert a profound influence across the entire brain by modifying the global brain state.

In the present work, we therefore systematically characterized the effects of ASG using a paired-tone paradigm with variable ISIs (125–2000 ms) and recorded brain responses of awake mice at a large-scale level with high-density EEG and intracerebral electrodes in multiple brain areas ranging from as early as ventral cochlear nucleus (vCN) to higher-order brain areas (anterior cingulate cortex; ACC). We also studied the effect of auditory and ASG processing at the level of brain states using clustering analyses of surface voltage topographies and pre-T2 brain activity recorded intracerebrally from multiple brain regions.

## Materials and Methods

### Animals

Twenty-six male C57BL/6J mice (two to three months old; Charles River) were recorded in this study. We recorded epicranial EEG in seven mice (three to five recording sessions per animal, population statistics were performed across subjects). Intracranial recordings were performed in 19 mice. One to three recording sessions from different brain regions were accomplished in each animal. In a few cases, a similar brain region was recorded twice in two different recording sessions but in slightly different sites. Population analyses are based on the number of recording sites. All experiments were in accordance with the applicable Swiss and European regulations on animal experimentation and were approved by the Ethics Committee on Animal Experimentation of the University of Geneva and the Veterinary Office of Canton of Geneva.

### Surgery

The animals were anesthetized in an induction box with a 2.5% isoflurane and were mounted in a stereotaxic frame with continuous delivery of 1–1.5% isoflurane to maintain the anesthesia. Artificial tears ophthalmic gel (lacryvisc; Alcon) was used to avoid corneal drying and the body temperature was monitored through a rectal probe connected to a closed loop heating pad. An incision was made on the skin and the skin was retracted. Subsequently, the positions of epicranial electrodes or intracranial electrode entry points were marked with ink. The skull was covered by a layer of glue (Loctite; Henkel). Once the glue was dried, a small patch with a diameter of around 500 μm was removed at each electrode location. A ring-like head-holder was fixed on the occipital and nasal bones using dental cement (Kaladent AG). The center of the ring was filled with silicon sealant (Kwik-Cast, World Precision Instrument). Animals were returned to their cage (single-housed post-surgery) and antibiotics (trimethoprime-sulfamethoxazole, Roche) and nonsteroidal anti-inflammatory and analgesic medicines (Ibuprofene, Vifor, and Paracetamol, Bristol-Myers) were added to their drinking water. The animals were allowed to recover from the surgery for at least 3 d before the training started.

### Training

The mice were positioned on a stereotaxic frame equipped to head-fix the animals using the head-posts on their head. The training was continued twice a day for 3 d preceding the recording day. Acoustic stimuli were synthetized digitally using Real-Time Processor Visual Design Studio (RPvdsEx v88, Tucker-Davis Technologies) and were generated by a RZ6 processor (Tucker-Davis Technologies). Calibration of the setup was performed by measuring the sound pressure level (SPL) emitted by the speaker when driven by the wideband noise. The animals learned to stay still while paired-tone paradigm presented pairs of tones (wideband noise; 0.1–12.5 kHz) separated by a variable ISI (62.5, 125, 250, 500, 1000, and 2000 ms). The stimuli were noise bursts of 10 ms long including a 3-ms rise and a 3-ms fall and were presented from a frontal speaker (Electrostatic Loudspeaker, ES1, Tucker-Davis Technologies) positioned at 20-cm distance from the center of the head. The ISI of 62.5 ms was not analyzed here as it was too short epoch for the most of analysis in the present study. The sound intensity, delivered from our calibrated sound system, was started at 40 dB and was increased on successive sessions of training until it reached 70 dB on the last day of training. This intensity was then used during every recording session. An intertrial interval between 8–12 s separated each pair of tones ensuring no remaining brain activity from the preceding trials at the beginning of each trial.

### Epicranial and intracranial electrophysiological recordings

**Figure 1.**
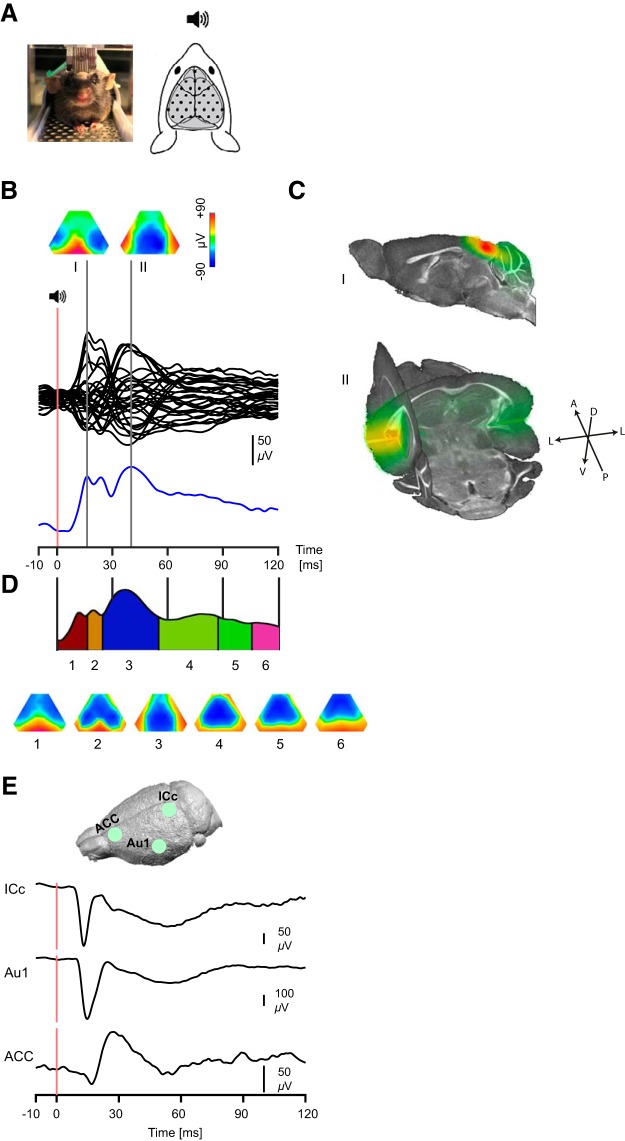
AEP in an example subject with epicranial recording. ***A***, Awake head-fixed animal during epicranial recording (left) and corresponding electrode placement map on the surface of the skull. ***B***, eAEP of an example subject following auditory sensory stimulation. The lower trace (blue) depicts the GFP of the shown EEG traces (average of 32 trials). The traces are time-locked to auditory stimulation (red bar at time 0) and two gray lines correspond to two GFP peaks. Voltage topographic maps at two time points with a peak GFP are shown at the top. ***C***, respective source localized maps of the two topographies shown in B suggesting localization of main generators in the inferior colliculus and auditory cortex. The upper map represents a sagittal section of ∼0.6 mm lateral to the midline. The axes of orientations for the 3D map is also provided. A, P, D, V, and L refer to anterior, posterior, dorsal, ventral and lateral orientations, respectively. ***D***, Clustering (top) of the auditory response shows a sequence of topographies (bottom) corresponding to the large scale network processing auditory response. The temporal extent of the identified maps appears as segments of the grand average GFP trace. Back-fitting of the clusters to individual animals’ ERPs validated the clustering analysis reproducibility across subjects as it showed monotonically increasing onsets and latencies of best correlation of each component map. Onset and latency differences between pairs of successive maps were all significant (*p* < 0.01). ***E***, Schematic representation of intracranial recording sites (top) and intracranial ERPs of example subjects recorded from the ICc, Au1 and ACC.

The details of surface recordings were described previously (Mégevand et al., 2008; Quairiaux et al., 2010). Briefly, the mice were anesthetized lightly for a short period and a grid of epicranial electrodes (32 electrodes) was lowered and adjusted to contact points on the skull (Fig. 1*A*). The brief anesthesia ensured precise adjustment of electrode tips such that they all have direct contact with the skull. The electrode tips were immersed in EEG paste (EC2, Grass Technologies) before contacting the skull yielding a final impedance of ∼50 KΩ. For intracranial recordings, an A16 probe (NeuroNexus) was positioned above the surface of brain at entry point and was lowered gently until it reaches the desired depth. Following anteroposterior (AP), mediolateral (ML), and dorsoventral (DV) coordinates relative to bregma, midline, and surface plane tangent to bregma were used for these recordings according to a mouse brain atlas (Paxinos and Franklin, 2004), respectively: –5.20, 1, and 2.25 for ICc; –2.7, 4, and 2.6 for primary auditory cortex (Au1); and 1.3, 0.2, and 2.4 for ACC. All recordings were performed from the right hemisphere and electrodes were laterally positioned such that a lateromedial angle of 20° guides the probe to the desired target. After the positioning of the surface grid or intracranial probes, the light anesthesia was removed, and the animals were allowed to completely wake up before the recording starts. Usually, the animals woke up within <5 min but an additional 15 min were allowed to avoid any effect of anesthesia on recordings. Paired-tone paradigm was run at the beginning of each recording session. A Digital Lynx SX (Neuralynx) data acquisition system was used for recording both surface and intracranial recordings. Sampling rates of 4 kHz (low-pass: 2 kHz) and 16 kHz (low-pass: 8 kHz) were used for surface and intracerebral recordings, respectively. To synchronize recordings and acoustic stimuli, digital triggers were received from the real-time processor RZ6 (Tucker-Davis Technologies) during all recording sessions. A thin reference pin was implanted in the cerebellum of the opposite hemisphere compared to recording site.

### Data analysis

Data analysis was performed using the Cartool software (D. Brunet, Center for Biomedical Imaging, University of Geneva, Switzerland; Brunet et al., 2011) and custom-written MATLAB functions. In this manuscript, auditory evoked potential refers to AEPs in general, eAEP refers to epicranially recorded AEPs and iAEP refers to intracranially-recorded AEPs, i.e., drawn from the local field potentials (LFPs).

#### Epicranial data analysis

Epicranial data were referenced against an average reference and downsampled to 1 kHz before any subsequent analysis. Global field power (GFP) is based on the spatial standard deviation of voltage values at all electrodes and represents the magnitude of activity (potential field) at a large-scale level (Lehmann and Skrandies, 1980). This measure was calculated according to the following formula:

$$GFP=\sqrt{\frac{\sum_{i=1}^{n}{\left(v_{i}-\bar{v}\right)}^{2}}{n}},$$

where *n* is the number of electrodes, *v* the voltage measured at electrode *i* and $\overset{¯}{v}$ is the mean potential across electrodes. GFP provides a single, positive and reference-free value reflecting the strength of neural responses recorded at all electrodes above all over the brain (Murray et al., 2008). The above formula was used to calculate the GFP for each time point from the grand average data. For each ISI, grand averages were calculated by averaging eAEPs of all animals. To represent the distribution of the surface potentials, we constructed topographic maps by interpolating voltage values between electrodes using Delaunay triangulation as described previously (Mégevand et al., 2008). Note that this interpolation is for representation and the clustering algorithm was applied on raw EEG. Topographical mapping is a reference-free measure (Geselowitz, 1998) that represents global configuration of the underlying neuronal activity. Furthermore, it has been shown that different topographies are necessarily generated by different populations of neurons (Vaughan, 1982; Srebro, 1996). To take advantage of these topographies even more efficiently, rather than constructing topographies of a fixed time windows, we used a two-step clustering analysis. First, we used a topographic hierarchical clustering algorithm (Brunet et al., 2011) to identify the most dominant topographic maps in the grand averages of eAEPs representing the distribution of field potentials on the surface. For each ISI, a 500-ms epoch starting from the onset of the T1 was used for the clustering analysis. In our analysis, we restricted the number of clusters to a maximum of 20 while we did not impose a minimum threshold such that it was allowed to lead to a single cluster. Each cluster had to include at least four consecutive time points (4 ms). This analysis clusters multivariate signals into a sequence of potential maps based on the spatiotemporal similarity of voltage values across time while preserving maximum variance present in the data. Each cluster map (topographic map) is thought to represent a particular configuration of active generators within the brain during which the voltage potentials on the surface are stable. This constellation of active brain networks is reflected in a particular brain surface topography and is defined as a brain state. Subsequently, we fitted the result of clustering back to the grand average of the response following the T2 and to the time series of each mouse to statistically verify the first step of analysis and to test whether the cluster maps are shared among individual mice. This step yields measures of presence, duration and power for each cluster map that can be statistically compared between different conditions.

#### Intracranial data analysis

Before any analysis, intracranially recorded data were low-pass filtered at 300 Hz and downsampled to 1 kHz. The low-pass filtered signal contains LFPs from which iAEPs were drawn. iAEPs were obtained by averaging across trials and grand averages were obtained by averaging across iAEPs of individual recordings. Latencies of iAEPs were calculated using the onset of the initial positive peak of the ICc and the onset of the main negative deflection of the Au1. For the ACC, the timestamp of the initial trough was considered as the latency. We used current source density (CSD) as a guide to the best electrode channel in each recording session. CSD was estimated as the second spatial derivative of the LFP along the depth as described earlier (Mégevand et al., 2008) according to the following formula:

$$CSD=\frac{V_{h-\Delta h}-2\cdot V_{h}+V_{h+\Delta h}}{\Delta h^{2}},$$

where *V* is the potential at position *h* and *Δh* is the distance between electrodes. CSD represents the spatiotemporal profile of extracellular current sinks and sources. To extract spectral characteristics of the LFPs, the signal was epoched containing segments that start 3 s before and end 3 s after each trial. To decompose the epoched LFP traces for frequency analysis, a family of complex morlet wavelets (ω) were used based on the following formula:

$$\omega_{f=}e^{i2\pi tf}e^{-t^{2}/(2\sigma^{2})},$$

in which i is the imaginary operator, t is time, f is frequency and σ represents the width of the wavelet. σ, the width of the wavelet, was defined as λ/(2πf) where λ is the number of wavelet cycles ranging from 4 to 10 (logarithmically spaced). The variable number of cycles was used to account for the trade-off between temporal and frequency resolution of the analysis. Example single units presented in this study were extracted by automatic spike detection and clustering using Klusta (Rossant et al., 2016) followed by manual sorting.

### Statistical analysis

Statistical analyses were performed using MATLAB and Prism software (GraphPad). All statistics that are presented in this study concern group-level statistics based on the averaged data (e.g., AEP, power, etc.) of individual animals or recording sessions. Paired *t* tests with Bonferroni corrections for multiple comparisons whenever applicable and one-way and two-way repeated measures ANOVAs followed by Tukey’s *post hoc* tests were used for comparisons among different conditions. For clustering analysis, the grand averaged data were first clustered followed by back-fitting to individual subjects. The results of fitting were then statistically tested using two-way ANOVAs. FDR corrected paired t-tests were used to determine a significant difference between GFP at individual timestamps compared to baseline GFP. Pearson correlation coefficients were calculated as a measure of signal similarity between different conditions. The specific contrasts and the statistical tests that have been employed to examine these contrasts are described wherever applicable.

## Results

### Large-scale mapping of AEPs in awake mice

We first recorded AEPs in awake animals using epicranial electrodes (eAEP) covering the entire dorsal surface of the brain (Fig. 1*A*). By considering the electric potential map at the surface of the brain, this method allows spatiotemporal analyses of the propagation of evoked activities at the large-scale level (Mégevand et al., 2008). As illustrated in Figure 1*B*, a single tone (wideband noise) evokes large voltage waveforms (eAEP) invading all electrodes above the brain. Voltage topographies at the peaks of the GFP curve shows that the activity is first dominated by a high amplitude positivity in the caudal region of the brain and later by high amplitude positivity in the lateral brain regions (Fig. 1*B*). These two regions of focal activity were localized at coordinates close to the inferior colliculus and the auditory cortices, respectively, which was confirmed by applying a source localization algorithm of the putative generators (Fig. 1*C*).

Although voltage topographies at individual time points could give insight into dominant brain processes at each time point, these processes often remain active for a period that encompasses several time points. to characterize the sequence of the large-scale brain processes following the auditory stimulation in more details, we clustered (Fig. 1*D*) the surface maps of the grand average eAEPs (*n* = 7 mice; first 120 ms) using a topographic hierarchical clustering algorithm (Mégevand et al., 2008; Brunet et al., 2011). The optimal number of clusters to describe the grand mean data were determined by integrating a number of clustering measures such as global explained variance, cross-validation and Krzanovski–Lai criteria (Brunet et al., 2011). Since different map configuration represent different underlying neuronal generators, the cluster analysis allows to determine the different brain processes activated by the stimulus. By fitting these cluster maps back to the data (using spatial correlation calculation and winner-takes-all labeling), the time period during which each of these maps are present can be determined (Michel et al., 2001 for a detailed description of the method). Importantly, this fitting procedure results in a segmentation of the evoked potentials into time periods within which a certain scalp topography remains stable, indicating a particular brain state during information processing. The reproducibility of the clustering and fitting procedures was validated by back-fitting of the clusters to individual animals ERPs. Each map was present in at least six out of seven animals and the sequential maps showed monotonically increasing onsets from map one to six. The differences of onsets and best correlation latencies between pairs of successive maps were all significant (*p* < 0.01 for all). As shown in Figure 1*D*, the first stable map lasted 17 ms and was dominated by a large caudal activity presumably corresponding to the first station of the ascending auditory pathway that can be detected on the dorsal surface of the brain, i.e., the IC as explained above. During maps 2 and 3 (between 18 and 25 and 25 and 54 ms post-T1, respectively), positive voltages become more lateral, surrounding a large area of relatively more negative potentials. This voltage configuration presumably reflects the curved lateral dipole orientations of the auditory cortical areas. Finally, maps 4–6 (between 55 and 80, 81 and 99, and 100 and 120 ms post-T1, respectively) show centro-frontal negativities that suggest a propagation of activity to the frontal regions (note that a significant focal brain activity can be reflected as local maxima or minima in voltage topographies). Therefore, this sequence of maps shows brain activities that likely correspond to main areas within the auditory pathway (i.e., inferior colliculus and auditory cortices) as well as higher order processing areas of prefrontal/anterior cingulate areas. Figure 1*E* shows the intracranial recording sites and ERPs of an example subject. As expected, the onset of the evoked potentials recorded in the inferior colliculus correspond to the onset of the surface potentials recorded in the caudal region of the EEG. Intracranial recordings also confirm that auditory stimulation induced significant activities in the frontal cortical region.

### Long-lasting sensory gating of EEG-recorded AEPs

**Figure 2.**
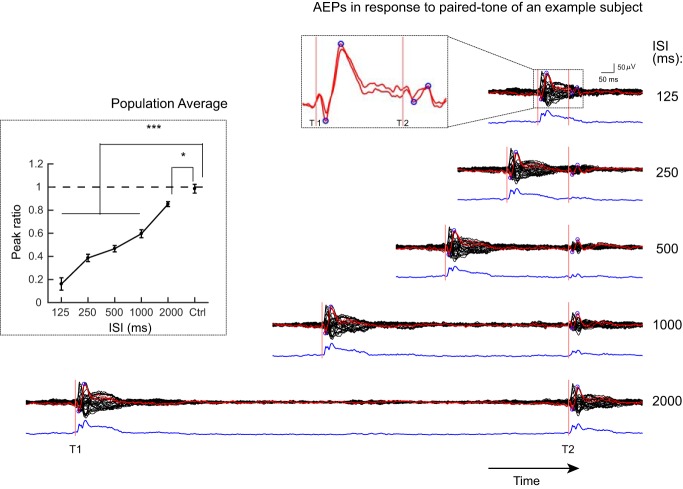
Epicranial EEG recording of sensory gating using paired-tone paradigm. The eAEP of an example subject (same as Fig. 1*B*) to a pair of tones with different ISIs are shown. The red traces correspond to left and right electrodes above the auditory cortex and black traces represent all other electrodes. The blue circles indicate the peak-to-peak amplitude used to quantify sensory gating. Top inset shows the zoomed segment (only electrodes above auditory cortices) within the dashed rectangle to highlight the strongly gated but still present response to T2 at the ISI of 125 ms. The blue trace in bottom of each EEG trace corresponds to the GFP. The attenuation (gating) of the response to the T2 is visually clear. The left inset shows average sensory gating ratios (*n* = 7 mice) quantified based on the eAEPs of auditory electrodes. Note that even at 2000 ms ISI, there is a significant sensory gating. The ratio equal to 1 means no gating and the smaller the ratios are, the stronger the gating is. Error bars indicate SEM; *p < 0.05, ***p < 0.001.

We then recorded surface EEG while paired auditory stimuli (wideband noise) were presented. The ISIs were randomly varied (125–2000 ms) across trials. As illustrated in EEG traces of an example subject in Figure 2, the eAEP and GFP amplitudes in response to the T2 are smaller as compared to the response to the T1 for all ISIs. This gating effect decreased with the duration of the ISI but was still present even at the longest ISI of 2 s. To quantify the effect of the duration of ISIs on the magnitude of sensory gating, we calculated the ratio of the peak-to-peak response magnitude of the T2 to that of the T1 at electrodes above the left and right auditory cortices (Fig. 2, left inset). A one-way repeated-measures ANOVA and *post hoc* Tukey’s tests for multiple comparisons showed that the ratios were significantly lower than control (*F*_(5,30)_ = 103.2; *p* < 0.0001) at all ISIs although the degree of gating was decreasing (i.e., bigger ratios) with increased ISIs. The ratio of the T1 responses of two randomly selected ISIs was used as control (minimum inter-trial intervals between two paired tones = 8 s). Note that T2 was still processed even at the shortest ISI (125) where the gating was the strongest, as it is visible from the small trough to peak component after T2 (Fig. 2, top inset). This was confirmed by a one-sample *t* test showing that the T2 response amplitudes were significantly larger than zero (*t*_(6)_ = 4.62, *p* < 0.01).

### Decreased amplitude but unaltered topography of T2-evoked brain states for long ISIs

**Figure 3.**
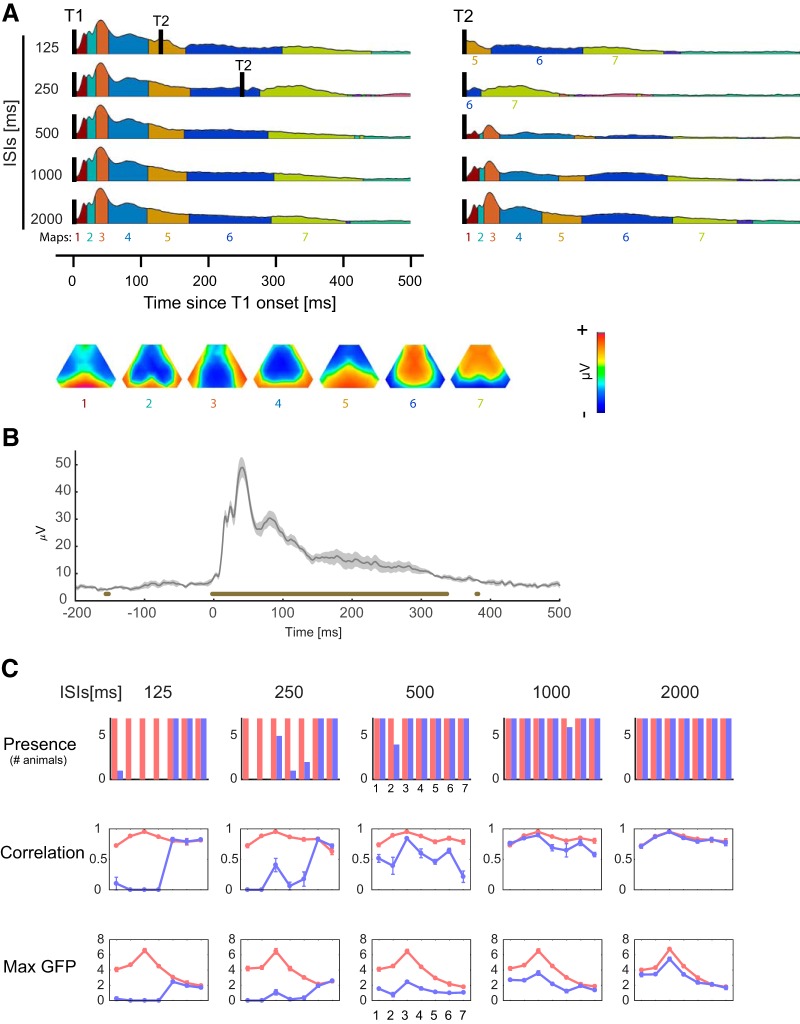
Similarity of functional brain states during post-T2 of short ISIs and time-matched period of long ISIs during which there is no T2 yet. ***A***, left panel, Result of the clustering analysis of the grand-average eAEP during the 500 ms period following T1. During this period, T2 takes place in short-ISI conditions but not in the long ISIs. The corresponding spatiotemporal topographic maps are presented below. Each map is labeled with the same color code that is used for the clusters. The result of clustering was fitted on the post-T2 epochs (right panel) revealing that the same sequence of brain states was activated in response to the T2 after long ISIs as compared to the sequence following the T1. For short ISIs, it shows that initial segments of T1 sequence were curtailed and suggests that the cluster sequences continue from where T2 stimulation enters in the timecourse. ***B***, GFP at a representative ISI (500 ms). The thick line indicates fdr-corrected significant enhancement of GFP compared to baseline (–200 to 0) power. ***C***, Quantitative fitting results of the clusters across individual subjects. “Presence” histograms show the number of the animals having a given topographic map. Note general similarity of presence, correlation and power of T1 (red) across different ISI conditions regardless of the presence or absence of T2 stimulation. This is also demonstrated by the convergence of correlation and power curves of T2 (blue) to those of T1 after maps 5 and 6 for ISIs of 125 and 250, respectively.

Although classic method of measuring ASG gives information on the magnitude of gating, it neglects dynamics of simultaneous brain activity in other brain regions. Therefore, to examine the effects of sensory gating on large-scale brain networks, we used the clustering analysis of the surface auditory evoked voltage maps described above. For this analysis, we first took epochs of 500 ms starting from T1 of the grand averages of the responses to T1 of all ISIs together. Figure 3*A* shows the result of this clustering (Fig. 3*A*, left panel) and the fitting of the resulting clusters on the grand average responses to T2 (Fig. 3*A*, right panel, 500-ms window from T2). The clustering of T1 grand average maps revealed a sequence of brain states similar to the one shown in Figure 1, indicating the propagation of activation across the auditory brainstem, the auditory cortex and the frontal brain regions. This sequence of brain states and its reproducibility was confirmed by back-fitting the clusters to the time series of individual animals: clustered maps were strongly stable across ISIs, as illustrated by the presence, correlation and max GFP parameters (Fig. 3*C*, red histograms and traces), even in the presence of T2 for the shortest ISIs (125 and 250 ms, see Resistance of T1-evoked brain states at short ISIs). The stability of the brain states faded after ∼350 ms post-T1 as no stable clusters were detected further on. This analysis suggests long lasting duration of stable brain states beyond initial AEP components determined in EEG or LFP traces. After this period (∼350 ms), the strength of overall neural response was reduced to the baseline level as illustrated by the GFP values that were not significantly stronger than baseline (–200 to 0 ms; Fig. 3*B*).

For the ISI of 500 ms and longer, fitting these clustered maps evoked by T1 to the grand average eAEPs evoked by T2 indicated a similar sequence of topographies (Fig. 3), although with a reduced power, illustrating the effect of sensory gating. Thus, for ISIs of 500 ms and longer, decreasing the amplitude of the responses did not alter the sequence of voltage topographies, i.e., the spatial organization of the evoked brain states.

### Resistance of T1-evoked brain states at short ISIs

**Figure 4.**
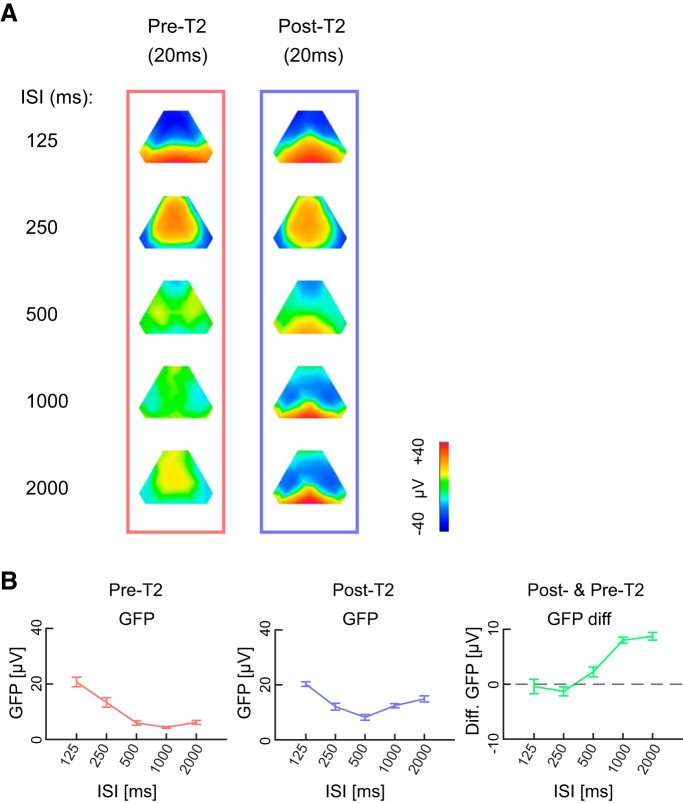
Similarity of pre-T2 and post-T2 maps at short ISIs. ***A***, Topographic maps of pre-T2 and post-T2 EEG (20 ms each) are shown separately for each ISI. At short ISIs, visually distinguishable similarities exist between pre-T2 and post-T2 maps. ***B***, Pre-T2, post-T2 and and the difference between them across different ISIs. As shown on the left panel, pre-T2 GFPs are higher for ISIs of 125 and 250. There is also approximately zero change in power from pre-T2 to post-T2 at these ISIs.

Importantly, the clustering and fitting analyses revealed an important characteristic of the brain states: the sequence of brain states were not perturbed by T2 stimulations of short ISIs (125 and 250 ms) that take place within the post-T1 period of the evoked brain states. As shown in Figure 3*A*, left panel, the brain states of short ISIs at the time of T2 stimulation are similar to time-matched post-T1 brain states of long ISIs during which there is no T2 stimulus yet. This was confirmed by the fitting of the clustered maps onto the grand average eAEPs at 125 and 250 ms (Fig. 3*A*, right panel) and by the back-fitting to the time series of individual animals (Fig. 3*C*, blue histograms and traces) that revealed the absence of the earliest maps but the presence of maps 5–7 for T2 of the short ISIs. The continuity of brain states regardless of T2 stimulation following short ISIs was also confirmed by a model free comparison of voltage topographies of a fixed period immediately preceding or following T2 without using any clustering analysis (Fig. 4). As shown in this figure, the difference between pre-T2 and post-T2 GFPs is also approximately zero for the short ISIs suggesting lack of change in the power of the ongoing brain activity. Thus, the brain networks that are active at the moment of T2 stimulus continue to stay active despite T2 stimulation. These results suggest that the dominant generators of the brain states are resistant to perturbation by the new stimulus at the short ISIs and that the AEP induced dipoles are shadowed by still robust post-T1 dipoles generating those brain states. Thus, these analyses not only show long lasting stability of brain states beyond initial AEP components, but also suggest that these states are resistant against perturbation by T2.

### Hierarchical enhancement of sensory gating

**Figure 5.**
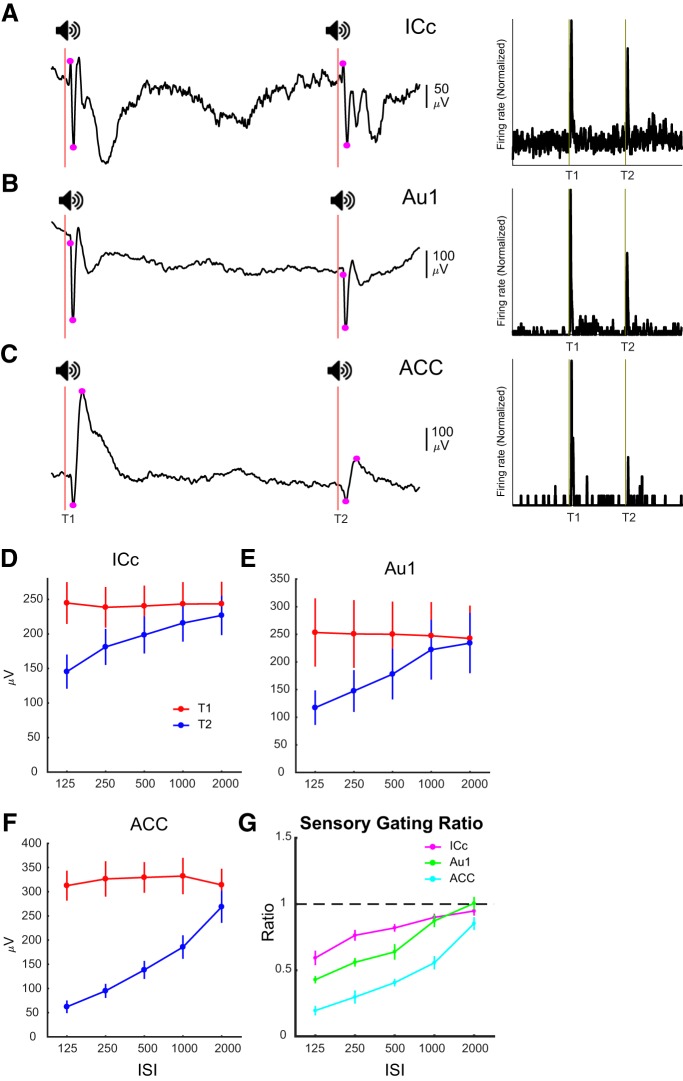
Sensory gating in intracranial signals recorded from ICc, Au1 and ACC. ***A–C***, Example iAEPs (left panels) and PSTHs (of all units recorded during a single session) recorded from the ICc, Au1, and ACC, respectively. Magenta dots represent peak-to-peak measurement of the first major ERP component at each region. Latencies were calculated using the onsets of the initial positive peak for the ICc, the main negative deflection for the Au1, and the initial trough for the ACC. ***D–F***, Amplitude of ERP components measured as indicated in ***A–C*** in each region, i.e., ICc (*n* = 12), Au1 (*n* = 11), and ACC (*n* = 10), in response to the first (red) and second (blue) tones. The larger the area between two curves is, the stronger the sensory gating is. ***G***, The ratio of sensory gating for different components. The gating increases progressively from the ICc through auditory ERP to the ACC ERP. Error bars signify SEM.

We then conducted intracranial recordings in ICc, Au1, or ACC (Fig. 5) to investigate local dynamics of brain processes with a greater precision. As expected, mean latencies of responses increase from the ICc region (*n* = 12; 6.43 ± 0.20 ms) to the Au1 (*n* = 11; 10.71 ± 0.24 ms) and the ACC cortices (*n* = 10; 15.69 ± 0.24 ms). Figure 5*A–C* shows example subjects’ iAEP (Fig. 5*A–C*, left panels) and peristimulus time histograms (PSTHs; Fig. 5*A–C*, right panels) in all three regions with a 500 ms ISI, illustrating the gating of T2 responses at the level of both iAEPs and single-unit firing. We computed the amplitude of iAEPs in all animals by measuring peak to peak magnitude of early iAEP components evoked by T1 and T2, as indicated in Figure 5*A–C*. Repeated-measures two-way ANOVAs on the amplitude of iAEP components (Fig. 5*D–F*) revealed significant main effects of both ISI duration (*F*_(4,44)_ = 14.51, *p* < 10^−4^; *F*_(4,40)_ = 16.84, *p* < 10^−4^; and *F*_(5,45)_ = 41.43, *p* < 10^−4^ for the ICc, Au1, and ACC, respectively) and tone (T1 or T2; *F*_(1,11)_ = 32.29, *p* < 10^−4^; *F*_(1,10)_ = 18.84, *p* < 0.002; and *F*_(1,9)_ = 86.14, *p* < 10^−4^ for the ICc, Au1, and ACC, respectively) and a significant tone × ISI interaction (*p* < 10^−4^). Bonferroni *post hoc* tests corrected for multiple comparisons revealed a significant decrease (*p* < 0.001) in the amplitude of T2 response at all ISIs except 2000 ms in the ICc and Au1 and 1000 ms in the Au1. In the ACC, there was a significant reduction at all ISIs including 2000 ms (*p* < 0.05 for 2000 ms and *p* < 0.001 for all other ISIs). The area between the T1 and T2 response curves is an indication of the magnitude of sensory gating (Fig. 5*D–F*); the two curves converge as the ISI increases, because of the reduction of gating at longer ISIs. We further quantified sensory gating at each region by dividing the amplitude of iAEP components of T2 to those of T1 (Fig. 5*G*). The first negative peaks in the ICc and Au1 were significantly gated at all ISIs but 2000 (*p* < 0.001 for the ISIs of 125, 250, and 500 and *p* < 0.05 for the ISI of 1000). In the ACC, the ratios were significantly different from the control at all ISIs (*p* < 0.05 for the ISI of 2000 and *p <* 0.001 for all other ISIs). Note that confirming surface EEG results, intracranial recordings also showed that the T2 is indeed processed even at the shortest ISI despite being strongly gated. This was statistically confirmed by one-sample *t* tests of ASG ratios for the shortest ISI that revealed significantly larger ratios than zero at all three regions (*p* < 0.001 for the ICc and Au1, *p* < 0.05 for the ACC). This confirms that the T2 was indeed processed across the hierarchy despite being strongly gated. In addition to the decreased magnitude of gating with increasing ISIs, the average T2/T1 ratios (Fig. 5*G*) illustrates another important aspect of sensory gating across different brain regions: the magnitude of gating progressively increases as we go from the auditory brainstem to the Au1 and ACC, suggesting a hierarchical enhancement of sensory gating. Two-way ANOVA (ISI duration and brain regions (ICc, Au1, and ACC) as factors) on the gating (T2/T1 ratios) with repeated measures on one factor (ISI duration) showed a main effect of the brain region (*F*_(2,30)_ = 40.92; *p* < 10^−4^) statistically corroborating the hierarchical enhancement of the ASG.

### Effects of the T2 on the LFP at the shortest ISI

**Figure 6.**
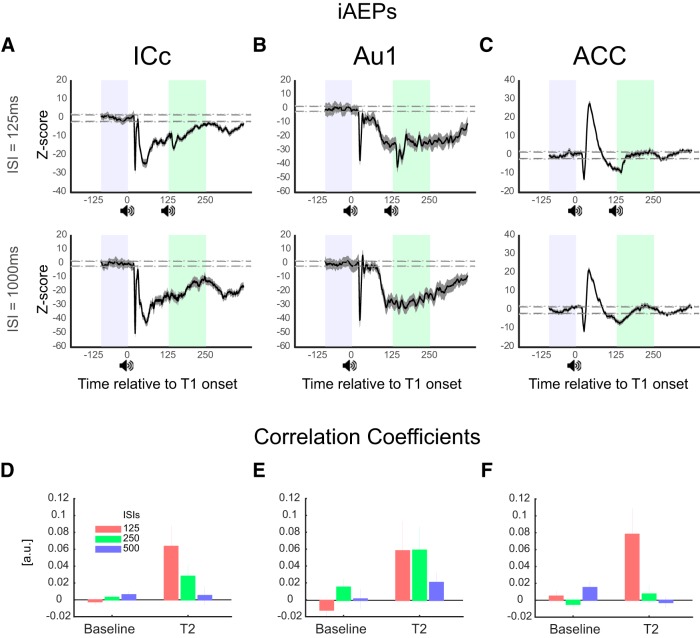
Similarity of post-T2 LFPs of short ISIs to time-matched LFPs of long ISIs. ***A–C*,** Show iAEPs of example recordings during 125 (top panels) and 1000 ms (bottom panels) ISIs. Highlighted periods represent pre-T1 baseline and period of interest (125–250 ms post-T1). Dashed lines represent Z-scores corresponding to *p* values of 0.05. ***D–F***, Correlation coefficient of LFP time series of post-T2 trials during ISIs of 125, 250, and 500 ms with time-matched (i.e., 125–250, 250–375, and 500–625 ms post-T1, respectively) period of 1000 and 2000 ms ISIs. This coefficient is based on the convolution of the signals and takes into account the temporal dynamics of the signal. As it is seen in the figure, post-T2 time series at short ISIs were significantly correlated to the time-matched period in long ISIs compared to baseline correlations. This correlation deteriorates as the ISI duration increases such that there is a non-significant trend at 250 ms ISI and the correlation coefficient is not different from the baseline at 500 ms ISI.

While local networks process T2 stimulus, the brain states remain unaltered for a period that encompasses short ISIs. Epicranial EEG revealed that the evoked brain states were resistant to perturbation by the new stimulus at the shortest ISIs, i.e., when T2 arrives during the evoked brain states. How this stability and resistance (against perturbation) of the brain states are reflected in the activity of local networks in the auditory pathway (ICc and Au1) and ACC? Might there be traces of stability in these signals although T2 stimulus is processed? We therefore further examined the similarity between short-ISI post-T2 iAEPs with time-matched long-ISI iAEPs. We calculated linear dependence between these signals using Pearson correlation coefficient. Coefficient of zero corresponds to absolutely inconsistent signals whereas coefficient of 1 corresponds to identical signals. Note that this measure takes into account signal trajectory across time and ignores scaling in the magnitude of the signal. Morphologically coherent signals across time will be highly correlated even if the amplitudes are markedly different. Therefore, this is an ideal measure to address whether T2 stimulation at different time points abolishes signal similarity or a degree of similarity survives despite T2 stimulation. Figure 6*A–C* shows visual similarity of iAEPs during the 350-ms window after T1 between a short ISI (125 ms) and a long ISI (1000 ms) conditions, i.e., with or without T2, of example recordings in the ICc, Au1, and ACC, respectively. Analysis of correlation coefficients between post-T2 epochs of short ISIs with the time-matched period of post-T1 epochs of long ISIs revealed a significantly higher similarity than pre-T1 baseline epochs for the ISI of 125ms (*p* < 0.001, *p* < 0.05, and *p* < 0.01 for the ICc, Au1, and ACC, respectively) supporting the extended brain stability suggested by the clustering analysis of the scalp EEG. There was a non-significant trend for the ISI of 250 ms, but there was no correlation for the ISI of 500 ms (Fig. 6*D*,*E*).

### Does pre-T2 activities can predict sensory gating?

**Figure 7.**
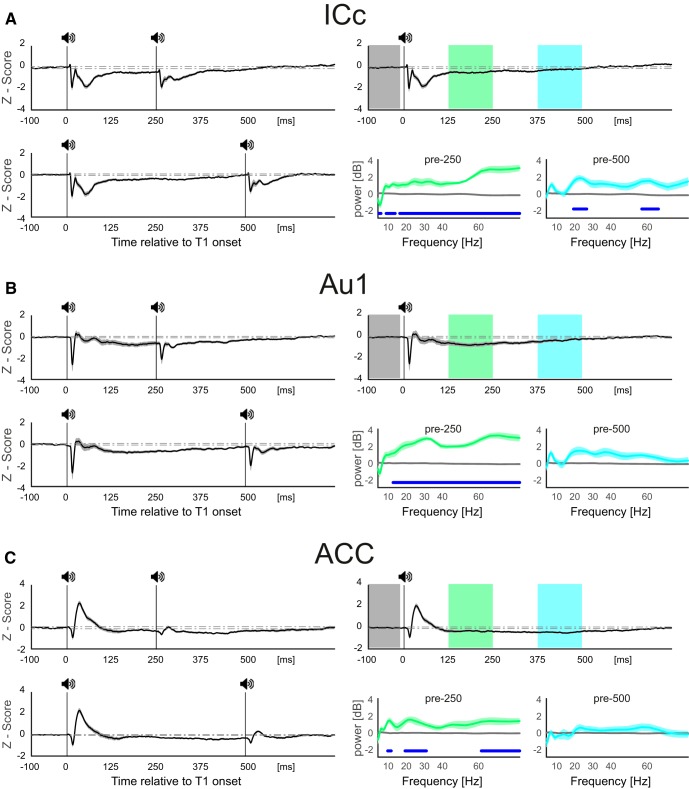
Pre-state dynamics following T1 and before T2 auditory stimulation. ***A–C***, left panels, Grand average ERPs across all recordings during ISIs of 250 (top panels) and 500 (bottom panels) for the ICc (*n* = 12), Au1 (*n* = 11), and ACC (*n* = 10), respectively. Right top panels, Corresponding grand average iAEPs calculated for the same time length relative to T1 as the left panels but without a T2, i.e., based on the 2000-ms-long ISI. The gray shades around iAEPs indicate SEM. Bottom right panels, Induced power levels during segments corresponding to pre T2 periods for 250-ms ISI (green highlighted segment in the iAEPs) and 500-ms ISI (blue segment), as compared to the baseline (gray segment). The thick blue lines indicate fdr-corrected significant difference between power of these periods compared to that of the baseline period.

Sensory gating is long lasting and the second stimulus is attenuated even after 2000 ms which is far beyond the remaining processes following T1 stimulation which lasts approximately up to 350 ms post-T1 (Fig. 3*B*). The brain states just before T2 may predict the ASG and therefore, pre-T2 signals in epicranial and intracranial recordings have to be studied to evaluate the role of pre-state dynamics on the ASG. As shown in Figure 4, the pre-T2 states, calculated from the epicranial EEG recordings, are different across different ISIs. GFP of these states are significantly different from baseline at short ISIs but not for ISIs of 500 ms and longer. These results indicate that the brain states we observed just before T2 does not explain the ASG. The iAEPs and power (induced) spectra of pre-T2 intracranially recorded LFPs are also depicted in Figure 7. As shown in Figure 7, left panels, it takes close to 500 ms for the iAEPs to reach steady state baseline levels. This is consistent with the previous analysis on the similarity of LFPs (Fig. 6) where we showed a significant similarity between post-T2 LFPs of short ISIs and time-matched post-T1 LFPs of long ISIs indicating that the traces of the response to T1 could be found after T2 when the ISI is short. Subsequently, we calculated power spectra of a long ISI condition (2000 ms) during baseline and during 125ms segments before time matching T2 of 250- and 500-ms ISIs (highlighted periods; Fig. 7, right panels) to avoid T2 response spectral leakage. The results of this analysis were consistent with the GFP computed from the epicranial recordings (Fig. 3*B*) and showed that the pre-T2 induced power was different from the baseline for 250 ms ISI but not for 500 ms ISI. Note that the power is significantly higher than the baseline up to 500 ms in the ICc. Taken together, we did not identify ongoing activity just before T2 for long ISIs that is significantly different from the baseline that could explain the gating of the imminent T2 response.

### ASG in vCN

**Figure 8.**
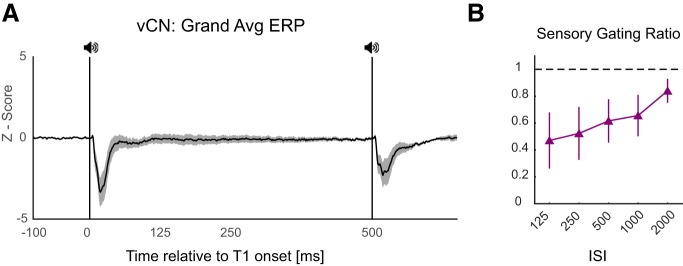
ASG at vCN. ***A***, Illustrates the grand average ERP during ISI of 500 ms recorded from the vCN of four animals. ***B***, Shows ASG (T2/T1 ratio) across different ISIs.

Despite the controversy about the origins of ASG, we showed that the ASG exists at the level of the ICc consistent with the indirect evidence (Malmierca et al., 2009), suggesting sensory-specific adaptation (SSA) at the ICc. Given this controversy and the lack of common understanding of the mechanisms of ASG, we recorded the activity of neurons at the entry of the auditory stimuli in the CNS in the vCN. The analysis of these recordings showed that indeed ASG already exists at the first station of auditory pathway in the CNS (Fig. 8). Note that the degree of gating in the vCN is similar to that of the ICc but we do not directly compare the gating in the vCN to other regions in the hierarchy since these recordings are based on fewer number of animals and we suggest a more comprehensive study of the role of vCN in the ASG.

## Discussion

We used variable ISIs and both epicranial and intracranial recordings to investigate large-scale brain networks governing ASG. We demonstrate that the ASG is long lasting, takes place in areas ranging as early as the vCN and ICc to higher order areas of Au1 and ACC and is hierarchically organized. Furthermore, we show that auditory stimulation results in the activation of a sequence of stable brain networks lasting up to ∼350 ms. During this period, the T2 (i.e., ISIs of 125 and 250 ms) of paired stimuli does not change the architecture of brain topographies evoked by the T1 suggesting that induced networks resist perturbation by incoming stimuli. This was evidenced by a striking similarity between voltage topographies representing active brain networks following T2 in short duration ISIs (i.e., 125 and 250 ms) and time-matched post-T1 topographies in long duration ISIs (i.e., 500, 1000, and 2000 ms) as well as a corresponding similarity between intracranially recorded LFPs. After this period, the brain reaches a baseline activity level and a second auditory stimulus induces similar network as the first stimulus. However, gating is preserved at least up to 2000 ms at the level of the amplitude of the neural responses.

Given the clinical relevance of ASG in which the degree of gating of the T2 is used as a pathologic biomarker of schizophrenia (Javitt and Sweet, 2015), most studies concerning ASG has used the same ISI as in the clinics, i.e., 500 ms (Kurthen et al., 2007; Rihs et al., 2013; Oranje and Glenthøj, 2014). At different timescales ranging from tens of milliseconds to a couple of seconds, distinct brain mechanisms might be responsible for gating of the response to T2 (Ulanovsky et al., 2004; Wehr and Zador, 2005). Our approach using variable ISIs in conjunction with epicranial and intracranial recordings enabled us to characterize ASG at global brain networks level and selected individual brain regions across a range of timescales. Although a few studies have used different ISI durations to study ASG (Wehr and Zador, 2005; Mears et al., 2006), these studies have usually been limited to one or two brain areas and not systematically characterized the effects of ISI duration on ASG. Our experiments revealed three characteristics of the ASG. First, there was still significant gating after an ISI of 2000 ms (longest in our experiments). Note that there was no detectable activity remaining from the T1 processing at long ISIs, and the GFP and local brain activity were similar to the baseline at ISIs of 500 ms or longer. Second, regardless of the mechanisms involved, there was a monotonically decreasing magnitude of ASG as the ISI increases (Figs. 2, 5). This suggests that either the mechanisms involved in the ASG at short intervals (e.g., synaptic inhibition) are stronger than those involved in longer ISIs or the mechanisms that act at longer ISIs are also active at short intervals and therefore there is a synergy between mechanisms acting at short intervals. Multiple local, bottom-up and/or top-down mechanisms may be responsible for ASG and deciphering their relative contributions needs further research. Based on our data, however, we can suggest (1) the fact that pre-T2 states were different across different ISIs and (2) could not predict whether the arriving T2 will be gated or not and (3) the fact that the T1-induced activity regresses back to the baseline level before 500 ms indicates that these mechanisms act earlier but their effects (e.g., synaptic depression) are long-lasting. Third, the dynamics of gating in the ICc, Au1 and ACC suggests a progressively amplified ASG across the hierarchy. Using ISIs ranging from 64–512 ms, Wehr and Zador (2005) suggested that it is unlikely that ASG in auditory cortical neurons at ISIs longer than 100 ms is inherited from earlier auditory brain areas and proposed that intracortical or thalamocortical synaptic depression is the likely mechanism of sensory gating at these intervals. In our experiments, the existence of strong gating at long ISIs in areas as early as vCN and ICc in the auditory pathway suggest that gating of cortical neurons is partly inherited from earlier brain regions. Although SSA has been demonstrated in the non-lemniscal regions of subcortical inferior colliculus (Malmierca et al., 2009), ASG at the level of inferior colliculus has not been clearly addressed. Our results demonstrate that indeed sensory gating takes place in the inferior colliculus centralis. This is consistent with studies on SSA, which also suggest a degree of adaptation in subcortical brain regions such as the ICc (Malmierca et al., 2009) and auditory thalamus (Antunes et al., 2010). Furthermore, unlike previous studies suggesting lack of SSA in vCN (Ayala et al., 2012), we showed significant ASG even at the level of vCN, which is the first auditory processing station in the CNS. While ASG and SSA are related phenomena in the auditory processing, there are differences in the paradigms used to study these two phenomena. Therefore, it is very likely that the discrepancy between our results and those of Ayala and colleagues emanates from the differences in the employed paradigms and indicates distinct characteristics of neural processing of auditory information. Our results showing sensory gating at the first station of auditory processing in the CNS enhance the plausibility of the hypothesis that the sensory gating is a basic property of the brain hardwired in brain network assemblies, without ruling out the possibility that subcortical ASG maybe be driven by top-down cortical projections to the brainstem. On the other hand, the fact that we demonstrate a progressively amplified sensory gating across hierarchy, suggests that further thalamocortical and cortical mechanisms such as synaptic depression and feedback regulation enhance the magnitude of gating in Au1 and particularly ACC. This suggests a link between higher cognitive brain areas and low-level sensory processing and highlights the translational importance of low-level sensory processing mechanisms for disorders of higher cognitive functions as proposed by Javitt and Sweet (2015).

What is the state of brain networks following auditory sensory stimulation? In other words, how the dipoles across brain regions are configured following the stimulus? The stability of brain states activated by the T1 suggests that the dynamics of the activity of all dipoles result in a reproducible sequence of brain states during which distinct subsets of those dipoles are simultaneously active. Topographic mapping is a reference-free measure that represents global configuration of the underlying neuronal activity (Geselowitz, 1998) and based on the laws of physics, changes in voltage topographies reflect changes in underlying generators (Vaughan, 1982). The clustering method that we used (Mégevand et al., 2008; Brunet et al., 2011) divides the potential maps into meaningful clusters during which there is no change in topographic maps. Each cluster represents a particular brain state and is generated by a network of roughly simultaneously active sources (Michel and Koenig, 2018). Importantly, the stability of brain states extend far beyond the initial evoked potentials and lasts as long as 350 ms even in the absence of a goal-directed behavior associated with the tone as was the case in our experiments. Thus, we suggest that brain dwells back in the baseline resting state after this sequence of activated brain states fades. Finally, the stability and reproducibility of the configuration of all brain dipoles was robust such that a second identical tone, presented during this period, was unable to bring about a change in the architecture of sequential brain states ensuing T1 (Fig. 3). Note that this resistance of brain states to perturbation by T2 is not due to the fact that T2 is not processed (Figs. 2, 5). Consistent with this brain network stability notion, a recent fMRI study among human participants showed that indeed brain networks are stable at individual participants’ level and task states only modestly influences brain networks (Gratton et al., 2018). Investigating local brain activity using intracranial recordings provides further support for the results discussed above. First, it demonstrates that T2 has been processed across the hierarchy (Figs. 5, 6*A–C*) despite being strongly gated. Second, it supports the global brain network stability by showing traces of similarity between post-T2 LFPs of short ISIs and LFPs of time-matched long ISIs. Linear dependence of these LFP segments suggest that indeed there is a significant similarity in the morphology of signals between post-T2 period of the shortest ISI and time-matched period of long ISIs. This supports the resistance of the brain states to perturbation at short intervals by showing common features in the brain signal (oscillatory or non-oscillatory) regardless of T2 stimulation at short intervals. We suggest it is possible that membrane potentials of subpopulations of neurons continue to reverberate resulting in the long lasting and stable responses. However, the exact cellular mechanisms underlying the stability and resistance (against perturbation) of brain responses demand further investigation.

Might the extended brain stability represent a fundamental property of brain function? Might it have a functional role? Our experiments suggest that the extended stability of brain states and their resistance against perturbation may result from two factors: The strength of global brain networks as evidenced by the GFP (Fig. 3*B*) and the strong sensory gating of T2 response during this period (Figs. 2, 5). These two factors are interdependent and both may contribute to the resistance of stable brain states against perturbation at short ISIs. Future studies should examine whether there is a causal relationship between these two. Extended brain stability may also represent a way for the brain to integrate the stimulus with other information available from internal and external sources and further interpret the stimulus significance after an initial and fast processing of the sound itself. Finally, it may involve attentional activation of brain regions to prepare for an upcoming event or to inhibit activated brain regions in case of discarding the stimulus.

## Synthesis

Reviewing Editor: Leonard Maler, University of Ottawa Decisions are customarily a result of the Reviewing Editor and the peer reviewers coming together and discussing their recommendations until a consensus is reached. When revisions are invited, a fact-based synthesis statement explaining their decision and outlining what is needed to prepare a revision will be listed below. The following reviewer(s) agreed to reveal their identity: Bernhard Gaese, Manuel Malmierca. Note: If this manuscript was transferred from JNeurosci and a decision was made to accept the manuscript without peer review, a brief statement to this effect will instead be what is listed below.

Both reviewers have found that the ms has been thoroughly revised and streamlined, now focusing on the most important results. As Editor, I fully agree.

Overall, this is now a much clearer story. In addition, rearranging the sequence of statements and figures (now: Figs. 4 to 6) helps to see what results are supporting the statements about adaptation separated from the results on stability of brain states. What is meant by “brain states” in this ms. has been clarified as well along the suggestions by the reviewers.

In the introduction section, the statements about auditory sensory gating (still a somewhat bulky term) is now also related to the literature about forward masking, forward inhibition, and adaptation describing the same phenomena, however mostly at different level of integration.

Several missing methodological details have been added to the methods section. The additional explanation about the different types of evoked potentials is very helpful.

As already mentioned above, the results section has been structured more clearly. Additional information on the clustering and generation of surface plots is now a more solid basis for several of the statements made.

Both reviewers did have some minor final suggestions but making these requested changes will not require additional review but only an Editorial check. Please clearly indicate how you have addressed the near trivial changes requeste (rebuttal and redlined version) and I will rapidly do my final check.

Reviewer #1

The authors might want to think about explaining the (rather general) term “resistance” better (page 18/l. 360). I would suggest to (always) use “resistance against perturbation” or, even better “stability” instead. It remains rather unclear if it is an advantage or a disadvantage to not respond to new stimuli.

DETAILS:

1) Figure 2: x-axis (time) -calibration bar for the traces is missing MINOR (noted as page/line) (8/133) .. animals learned .. (8/139) .. sound intensity started at 40 dB SPL and was increased for successive sessions of training up to 70 dB SPL on the ...(( I guess you meant a calibrated sound system )) (9/157) (( How can the rostro-caudal, i.e. the AP-coordinate for Au1 be at -2.7. This is probably wrong, if coordinates for the two others structures are noted correctly )) (12/213) ...series of each mouse to statistically verify the first step ...... (13/237) ....(logarithmically spaced). The ..

Reviewer #2

The results and discussion somehow state that ASG is similar to SSA. This is not totally true, the type of stimulation is SSA requires to use the oddball paradigm therefore I would tone done a bit this comparison. Also, this may explain why authors show ASG in VCN. Ayala and colleagues found no SSA in VCN because they mostly recorded from DCN and again the stimulation is not similar. Please make a comment on this. Also, SSA is mostly found in non-lemnical regions of the subcortical IC and MGB. Not sure if this is the case also for ASG. Please comments on this and cite papers on this issue if necessary.

## Author Response

Synthesis Statement for Author (Required):

Both reviewers have found that the ms has been thoroughly revised and streamlined, now focusing on the most important results. As

Editor, I fully agree.

Overall, this is now a much clearer story. In addition, rearranging the sequence of statements and figures (now: Figs. 4 to 6) helps to see what results are supporting the statements about adaptation separated from the results on stability of brain states. What is meant by “brain states” in this ms. has been clarified as well along the suggestions by the reviewers.

In the introduction section, the statements about auditory sensory gating (still a somewhat bulky term) is now also related to the literature about forward masking, forward inhibition, and adaptation describing the same phenomena, however mostly at different level of integration.

Several missing methodological details have been added to the methods section. The additional explanation about the different types of evoked potentials is very helpful.

As already mentioned above, the results section has been structured more clearly. Additional information on the clustering and generation of surface plots is now a more solid basis for several of the statements made.

Both reviewers did have some minor final suggestions but making these requested changes will not require additional review but only an Editorial check. Please clearly indicate how you have addressed the near trivial changes requeste (rebuttal and redlined version) and I will rapidly do my final check.

We thank the editor for positive evaluation of our manuscript. We thank the editor and both reviewers for the comments, which we believe improved our paper substantially.

Reviewer #1

The authors might want to think about explaining the (rather general) term “resistance” better (page 18/l. 360). I would suggest to (always) use “resistance against perturbation” or, even better “stability” instead. It remains rather unclear if it is an advantage or a disadvantage to not respond to new stimuli.

We now use “resistance against perturbation” wherever we had used “resistance” alone.

DETAILS:

1) Figure 2: x-axis (time) -calibration bar for the traces is missing

We have added this now.

MINOR (noted as page/line)

Thanks to the comment of the reviewer, we have implemented the following minor changes.

(8/133) .. animals learned ..

(8/139) .. sound intensity started at 40 dB SPL and was increased for successive sessions of training up to 70 dB SPL on the ... (( I guess you meant a calibrated sound system ))

(9/157) (( How can the rostro-caudal, i.e. the AP-coordinate for Au1 be at -2.7. This is probably wrong, if coordinates for the two others structures are noted correctly ))

(12/213) ...series of each mouse to statistically verify the first step ...... (13/237) ....(logarithmically spaced). The ..

-

Reviewer #2

The results and discussion somehow state that ASG is similar to SSA. This is not totally true, the type of stimulation is SSA requires to use the oddball paradigm therefore I would tone done a bit this comparison. Also, this may explain why authors show ASG in VCN. Ayala and colleagues found no SSA in VCN because they mostly recorded from DCN and again the stimulation is not similar. Please make a comment on this. Also, SSA is mostly found in non-lemnical regions of the subcortical IC and MGB. Not sure if this is the case also for ASG. Please comments on this and cite papers on this issue if necessary.

We agree with the reviewer that while SSA and ASG are related phenomena, they are not the same. Different paradigms are used to study these phenomena and they address related but distinct characteristics of the neural processing of auditory information. We didn't aim to imply that they are the same but it is true that the way discussed SSA and ASG together was not completely clear. To clarify this, we added further explanation to the relevant part in the discussion (Page 24-25) which reads as following now (new text is colored):

“In our experiments, the existence of strong gating at long ISIs in areas as early as vCN and ICc in the auditory pathway suggest that gating of cortical neurons is partly inherited from earlier brain regions. Although SSA has been demonstrated in the non-lemniscal regions of subcortical inferior colliculus (Malmierca et al., 2009), ASG at the level of inferior colliculus has not been clearly addressed. Our results demonstrate that indeed sensory gating takes place in the inferior colliculus centralis. This is consistent with studies on sensory specific adaptation (SSA) which also suggest a degree of adaptation in subcortical brain regions such as the ICc (Malmierca et al., 2009) and auditory thalamus (Antunes et al., 2010). Furthermore, unlike previous studies suggesting lack of SSA in vCN (Ayala et al., 2012), we showed significant ASG even at the level of vCN, which is the first auditory processing station in the CNS. While ASG and SSA are related phenomena in the auditory processing, there are differences in the paradigms used to study these two phenomena. Therefore, it is very likely that the discrepancy between our results and those of Ayala and colleagues emanates from the differences in the employed paradigms and indicates distinct characteristics of neural processing of auditory information. Our results showing sensory gating at the first station of auditory processing in the CNS enhance the plausibility of the hypothesis that the sensory gating is a basic property of the brain hardwired in brain network assemblies, without ruling out the possibility that subcortical ASG maybe be driven by top-down cortical projections to the brainstem.”
